# Lifestyle Matters Randomized Controlled Trial of a Preventive Health Intervention for Older People: Qualitative Sub Study with Participants and Intervention Facilitators

**DOI:** 10.2147/CIA.S232108

**Published:** 2020-02-20

**Authors:** Gail Mountain, Kirsty Sprange, Robin Chatters

**Affiliations:** 1 School of Health and Related Research, Faculty of Medicine, University of Sheffield, Sheffield, UK; 2 Faculty of Medicine, University of Nottingham, Nottingham, UK

**Keywords:** preventive health intervention, older people, qualitative study, randomized controlled trial

## Abstract

**Objective:**

This qualitative study embedded within a randomized controlled trial was conducted to explore the acceptability, experiences of, and short-term impact of a preventive health intervention (Lifestyle Matters) from the perspectives of those who took part, and to uncover any evidence for the theorised mechanisms of action (improved participation and self efficacy) underpinning the intervention. It was also conducted to help explain the quantitative trial results.

**Methods:**

A purposive sample of 13 trial participants who had been randomized to receive the Lifestyle Matters intervention (approximately 10%) were individually qualitatively interviewed immediately following their involvement. All four intervention facilitators were also individually interviewed.

**Results:**

Evidence of the hypothesized behavioural changes could be identified within the interview data, demonstrating the potential of this intervention. However, lack of adherence to the overall intervention eroded receipt of benefit. This finding complements the quantitative trial results which found that the study had failed to recruit those who considered themselves to be at risk of age-related decline.

**Conclusion:**

This form of preventive health intervention requires proactive identification of those who recognise the need to make lifestyle changes. This is difficult if reactive health and social care systems are the main referral routes. The methodological approaches taken towards the study of complex interventions requires reconsideration if potential benefits are to be accurately assessed.

**Clinical Trial Registration:**

ISRCTN67209155

## Background

Preventive health interventions to promote health and well-being in the extended lifespan are a priority across the globe.^1^ The Lifestyle Redesign™ intervention improved the health and wellbeing of older adults who were living in sheltered accommodation and at risk of health disparities in one US study,^2^ and that of older adults attending some form of community provision in a second.^3^ The Lifestyle Matters intervention was inspired by Lifestyle Redesign™ and adapted to the UK context.^4^ Similar to the US intervention, Lifestyle Matters sought to improve health and well-being of older people at risk of decline, using the principles of occupational therapy and occupational science.^5^ Based on the findings from the US studies^2^,^3^ and UK feasibility study,^6^ multi-component preventive interventions such as those provided through Lifestyle Matters are recommended in UK guidance,^7^ with the guidance having a specific emphasis upon the benefits of social connectedness.

The Lifestyle Matters randomized controlled trial (RCT) measured intervention clinical and cost-effectiveness^8^ with 288 people aged 65 years and over. Participants living in one city in the north of England and in rural North Wales were recruited between August 2012 and April 2013 via General Practitioner (GP) mail outs to those who met the age criteria for involvement. Other recruitment methods included community engagement and health-care professional referrals, but these were in the minority. The recruitment process had to be pragmatic to reach the target number of participants within the trial timeline. Participants were randomly allocated to receive either the intervention or usual care, in couples (if both consented to participate) or individually.

Those randomized to the intervention were invited to take part over 16 consecutive weeks,^8^ which involved attendance at weekly, facilitated groups at local community venues with up to 12 others. Participants were encouraged to explore and enact activities of relevance to them from a menu of different topics, with community engagement being a crucial component. Examples of topics that participants could select from included the importance of activity for health, maintaining physical and mental well-being and safety in the home and community.^4^ The group could also suggest other topics not included in the manualized intervention. Additionally, monthly 1:1 sessions were included to enable participants to identify and work towards individual lifestyle goals, supported by a facilitator and using the knowledge and confidence gained from the group.^4^

Two facilitators for each site received a two day shared training and were supervised weekly by a senior occupational therapist at site. All were paid at Grade 4 (UK NHS Agenda for Change) rates.

Intervention theory is located in promotion of self-efficacy.^10^ It also takes account of a model, which proposes that occupational performance is determined by a transactional relationship between the person, their environment and the activities they undertake.^11^ The emphasis of the intervention and how it is delivered is located in participants’ identifying their own goals, sharing strengths and skills with others and provision of support required to overcome psychological barriers and enable practice of new or neglected activities in the community. Didactic sessions relevant to the needs of the specific group are woven into the programme to enhance knowledge of how to overcome the barriers to active engagement. It was hypothesised that this combination of approaches, including the positioning of the older person as the expert would facilitate both attitudinal and behavioural change.

How the different aspects contained within the intervention map on to the elements described within the underpinning theories are shown in Table 1. The various components contained within the manualized intervention are shown in the vertical axis and the elements within each theory on the horizontal axis.

**Table 1:** Intervention Theories and How They are Embedded Within the Intervention

Theory Elements	Bandura				Law		
	Mastery	Vicarious Experience	Verbal Persuasion	Positive Emotional States	Person	Environment	Activity
**Intervention elements**							
Group membership		X	X	X	X	X	
Community location and focus		X		X		X	X
Group sessions	X	X	X	X	X	X	X
1:1 sessions	X		X	X	X	X	X
Decision making and leadership	X	X	X	X			
Sharing experiences skills and solutions	X	X	X	X	X		
Enacting new and neglected activities with support from others	X	X	X	X	X	X	X
Didactic sessions		X	X			X	
Identifying/making lifestyle changes	X	X	X	X	X	X	X
Sustaining lifestyle changes	X	X	X	X	X	X	X
Positive coping with illness and age related disability	X	X	X	X	X	X	X

**Note:** X = intervention component maps on to theory.

Qualitative evidence can illuminate how individuals experience and perceive interventions.^12^ It can also help to explain quantitative findings.^13^ The primary RCT outcome was the mental health component of the SF-36,^14^ which was not significantly different between the control and intervention groups at either 6 or 24 months.^15^

Overall impact of the intervention assessed by interviews at 24 months post randomization with the same trial participants and those who were also randomized to the control arm has been reported.^16^ Despite this, it was considered that a shorter term analysis of the experience of the intervention would be beneficial in order to elicit participants’ immediate reflections of their involvement in Lifestyle Matters. In addition, intervention facilitators’ views of the intervention were obtained following intervention cessation to consider how delivery might be improved.

## Aim

The aim of these interviews was to understand the acceptability, experiences of and short term (6 months) impact of the Lifestyle Matters intervention upon participants and intervention facilitators, and consider the findings firstly within the context of our theoretical framework and secondly within the main trial results.

## Methods

The reporting of this study conforms to the Consolidated Criteria for Reporting Qualitative Research (COREQ) guidelines.^17^ A deductive framework approach was employed for analysis.^18^ The experiences of participants are described within the context of the hypothesised theoretical underpinning as shown in Table 1. We applied methodological rigour, viewing the quantitative and qualitative data sets as being complementary, undertaking purposive sampling, interviewing at two time points and detailing our analyses.^19^

### Participant Sampling and Recruitment

Trial participants allocated to receive the Lifestyle Matters intervention were purposively sampled upon cessation of their involvement in the intervention. Twenty potential participants were identified to represent differing rates of adherence to the intervention across the two sites (thereby ensuring comparability with the overall study sample; 19) and all were approached via mailed letter, which included study information. Other variables used to select participants for interview included living situation, education, occupation, and career type.

Thirteen consented to take part and seven decided not to, with reasons being unavailable (n=1), other commitments (n=1) and not being willing to take part (n=5).

All four intervention facilitators were approached via email following intervention cessation and all consented to participate in an individual interview at a venue of their choice. Written informed consent was obtained from all participants.

### Interview Process

Individual interviews took place in the home of each consented participant with duration ranging between 14 and 71 mins. The interviews of shorter duration were with those participants who were less invested in the intervention. This data was used so that the voice of these individuals could be represented as well as those who had been more committed. Facilitator interviews lasted between 55 and 95 mins.

Two interview topic guides for participants and facilitators, respectively, were derived from best evidence and researcher knowledge, particularly from the feasibility study.^6^ Feedback on draft guides was sought from the study PPI representative.

Interviews were conducted by a senior experienced post-doctoral female co-applicant (SC), and a second female with a Masters degree employed on the study (co-author KS). Neither had met any of the interviewees prior to the interview. The topic guide was adhered to but prompting also occurred. Data saturation was discussed at regular intervals and interviews ceased once saturation was reached (more participants would have been recruited if this had not been identified). Transcripts were returned to both participants and facilitators for comments and corrections.

### Transcript Coding and Analysis

A small number of transcripts were coded to refine the initial analytic framework, which was then applied to the same two transcripts by the researchers who also conducted the interviews to ensure comparable coding. All transcripts were subsequently coded and analysed using NVIVO 10 software. Matrix charts created out of the analysis were examined for cross cutting themes and patterns to inform the final interpretation. Quotes were selected from the analysis to illustrate the breadth of opinion and differences in emphasis, particularly between participants and facilitators. The study PPI representative provided feedback on the presentation of findings.

### Characteristics of Those Interviewed

As Table 2 shows, the ages of interviewed participants ranged from 65 to 90 years, thereby including different generations of older people. There were more females than males and those living alone predominated. Adherence to the intervention varied across the group. As can also be seen from Table 2, one person had only attended one group session but the majority had attended 10 or more. It can also be seen that adherence to the 1:1 sessions was poor; no one interviewed had received all four 1:1 sessions as required by the manualized intervention.

**Table 2:** Characteristics of Interviewees

Psueudonym	Age	Gender	Age Completed Education	Occupation	Living Situation	No Groups Attended	No 1:1 Attended	Total Sessions
May	68	Female	15	Skilled (non-manual)	Alone	10	1	11
Denise	70	Female	15	Unskilled	With another	9	0	9
Susan	69	Female	15	Unknown	Alone	14	2	16
John	92	Male	14	Skilled (manual)	Alone	9	2	11
Liz	79	Female	20	Professional	With another	1	0	1
Fred	72	Male	15	Partly skilled	With another	15	1	16
Peter	72	Male	22	Managerial/Technical	Alone	12	1	13
Julie	69	Female	15	Skilled (non-manual)	Alone	14	3	17
Marion	73	Female	26	Professional	Alone	14	3	17
Trevor	65	Male	18	Managerial/Technical	Alone	15	3	18
Alan	77	Male	15	Partly skilled	Alone	15	1	16
Betty	88	Female	14	Unknown	Alone	12	1	13
Mavis	77	Female	15	Skilled (manual)	With another	13	1	14

Following open advertisement, two female facilitators were recruited, trained, and supervised throughout intervention delivery at each site. For the North of England site, one was a social worker and the second a qualified occupational therapist. For the North Wales site, one was a community worker (also Welsh speaking) and the second a qualified occupational therapist. The ages of three were between 25 and 35, with the fourth, the community worker being in her 40s. To preserve anonymity we do not identify these individuals further in our report of study findings.

## Results

The views expressed by both participants and facilitators are described below. All participants gave examples of the factors that had mediated and moderated their engagement in the group element of the intervention.

### Intervention Characteristics

The facilitators noted the differences between Lifestyle Matters compared to other, more didactic groups. They described seeking to give control to participants, encouraging peer support and education and tailoring the intervention to the needs of the specific group.

It’s kind of like being more open, not stringent about the session plan, but being far more flexible and actually taking your lead from who are these people? Who is this group? Picking up from them and adapting the material you know. (Lynne; facilitator)

### Views of the Group Intervention

Facilitators reported that the most popular group topics were activity and health, physical and mental wellbeing, roles and routines, being stereotyped, safety in the community, food and nutrition and relaxation. Groups seldom focussed on friendships, spirituality and slips, trips, and falls.

Some groups identified additional topics from those in the manual including living on a pension, volunteering, local history and your community and how it has changed. Some topics stimulated more in-depth discussions, eg, health conditions such as dementia.

Diversity of group preferences was observed, eg, a poetry activity exploring stereotyping of older people worked well with one group.

They were really thrilled with what they’d produced and the end result and it set off people bringing in poetry and it was a really creative thing. (Chris; facilitator)

Whilst with another group the poetry;

….went down like a lump of lead. (Sarah; facilitator)

Facilitators spoke about needing to be aware of the potential sensitivity of some topics, eg, mental health and memory problems. They had to be able to adapt sessions to individuals and to the group and its dynamics.

### The Importance of Group Facilitation

Participants observed that group sessions were open, friendly and welcoming and that time had been made for everyone.

We, it certainly been encouraged within the group for everybody to have their say, to sort of lead the conversation … even some of, some of the quieter er ladies came out of their shell after a few weeks, because given the encouragement and stimulation this is what happens, yeah. (Fred)

How the facilitators enabled groups to choose which topics to explore was appreciated.

Facilitators ran it extremely well and they did open it up very much so that it became the group’s decisions rather than their decisions, er, which was very good. (Peter)

It was noted that the facilitators appeared genuinely interested in older people.

They really were, you could see, they really were interested and they put their heart and soul into it and you could talk to them about anything you know. (Denise)

Group dynamics were inevitably influenced by factors such as participant personalities, group size and peoples’ different experiences and stories.

….you know you can’t underestimate just how complex that is because you’re treading a very fine line between making people feel safe and able to talk about things and maybe being a bit more challenging making sure that everybody in the room is getting a chance to speak, and making sure that things are going all OK and that people feel alright and keeping an eye on the kind of emotion in the room and the pace of activities, is it suiting everybody? (Chris; facilitator)

The behaviour of some group members could be challenging at times as illustrated by this example;

Initially the group seemed to be treading on egg-shells a bit but then realised that this is who she is and just they, not accepted her so much but accepted her ways and I think a lot of people felt a bit sorry for her rather than scared of her so they had that sort of attitude change. (Debbie; facilitator)

One participant identified herself as being a challenging individual who did not wish to make relationships within anyone in the group.

Sharing experience was irrelevant because they did not share; they had totally different lives; none of them were university people. (Marion)

### Transport and Venue

Participants are required to arrange their own transport; an on-going challenge in North Wales.

… you are very limited as to what you can actually do, so it makes it difficult to get out and meet people and so on. (Peter)

Several participants described using community transport, or accompanying each other on public transport. This reportedly helped people to regain confidence when travelling.

The group venue was observed by facilitators as being integral to health and activity. Locally based amenities could be very accommodating, wanting to actively promote what they could offer.

They made time for us they gave us a speaker they let us go round their gallery they opened up the place to us, they are busy and they did that you know I think you know we’ve been very lucky with people in the community and community groups have been very helpful. (Lynne; facilitator)

### Perceived Intervention Benefits

Activities participants had engaged with were wide ranging; eg, diet and nutrition, computer sessions, painting, visits to local archives, museums, libraries, local monuments, and steam railways. Participants had arranged speakers, eg, the police, a credit union representative, pharmacists, and auction house valuators.

I mean one of the fellas in the group he was in his 70’s but he’s a volunteer on the welsh highland railway … He organised a block booking for us on the train, we had 12 seats reserved on the train … And that was a really good day I really enjoyed that … (Trevor)

It was evident that all participants understood that activity was an important part of the intervention, whether through providing opportunities to try new things, re-engaging in neglected activities or assisting those who were disconnected and isolated to get involved. Participants also described the activity they experienced through the groups as doing something differently to what they did before.

OK, Thursday, we’ve enjoyed it so much, why don’t we go out and make Thursday an activity day’. We’ve nothing else to worry about, we’ve no dependents as such, we can go, go out any day, but Thursday ‘cause we’ve got into a routine, ‘yeah, let’s go and try so-and-so. (Fred)

With the support, it was possible to find the impetus to pursue one or more activities or interests.

It’s opened more doors from a leisure aspect. (Mavis)

Several described how the intervention enabled them to understand that they could still take part in activities that they had set aside due to age or physical health. Even those who were active described how the intervention helped them to persevere, when previously they may not have bothered.

I might go along to the sports hall and see if anyone does Tai chi there … I thought it was a bit stupid at the time, but afterwards I’d go home and … ‘ah, bloody muscles’ [laughs], you know, and I have said once all this is over and the summer holidays are over I’ll call in because I used to go to the gym and go on the bike and that, you know, so I might try the Tai chi after it was brought up there, you know. (Alan)

Participants highlighted how activities were made interesting and fun by facilitators.

Yes then there was the one on diets … it was well it was interesting well a bit of fun as well. (John)

However, several also talked about not enjoying some activities, either due to lack of interest or pre-existing knowledge.

Enjoyment could be more motivating than awareness of the benefits of activity but the importance of both was appreciated.

Er I did see that it would promote health and recovery in people who’d perhaps been ill and people who don’t do much in the way of activities, it would get them up and exercising, which is good. (Fred)

Facilitators described how some people who were initially reluctant could become enthusiastic.

Another lady I’ve done a 1-1 with in that group … I can see changes in her as well … she was very very cautious and very quiet and very reticent and she has blossomed and is definitely wanting to engage in activities with other people. (Lynne; facilitator)

Awareness of the need to change unhelpful and sometimes unhealthy routines was fostered.

I have been a creature of habit with a programme like a robot to a realisation to whatever I’m doing can be done at any time of day it doesn’t have to be part of a set programme so er there’s certain amount of freedom yeah. (Mavis)

Participants’ resourcefulness was noted by the facilitators.

At the beginning the members were just so enthusiastic and they came up with the idea of bringing books they’d read in to put on a table for the tea and coffee break and to swap them, and that was from the word go, they were thinking of what, what can we do to share stuff you know. (Lynne; facilitator)

However, some individuals remained unable to recognise their own contribution.

We really did have a laugh, we had a good laugh but I don’t know that I contributed anything … Oh I gave them some information. I took some leaflets about things, you know, but well you don’t think that, do you? That you’re contributing, you just do it, you just take things. (Denise)

Facilitators observed how participants began to challenge stereotypes and prevailing expectations. Through the support of the group, one person with an overly caring family had successfully arranged her own holiday away. The intervention also enabled the “internalised stereotyping” to be challenged such as that expressed by the following participant.

I was significantly different socially and intellectually and motivationally and so on from the other people who were there. (Marion)

### Interpretations of the Individual Sessions

Facilitators all described the potential value of the individual sessions.

… getting to know people better and people used (the individual sessions) in lots of different ways really just for, it could be anything from feeding back about the group and picking up something that had happened in the group, was useful for me for kind of relationship building and just looking at support systems and just reinforcing that kind of group experience. (Chris; facilitator)

However, they also struggled to both explain and get engagement with individual sessions. This was rationalised in part by the focus upon group organisation during study recruitment but also because individual sessions could be potentially problematic.

I don’t know whether it veered on the, kind of, the patronising and intrusive, you know, like, well this is what, they’d agreed to come to a group, in their head, this is what they thought it was. (Lynne; facilitator)

It was described how those who saw themselves as outgoing, busy people with active lives said that they did not need individual sessions as they had no personal issues or goals that they wished to discuss.

This lack of clarity was reflected by participants with two of the 14 interviewed having no memory being offered individual sessions.

### Meeting New People, Belonging and Making Friends

Recruiting a group of strangers resulted in participants meeting new and different people.

It’s been, it’s been a pleasure to go and meet that group of people, a cross-section of people I didn’t know. (Fred)

This could bring opportunities for new learning:

Seeing different people getting to know what they do and you know it’s you know it you like alone but you don’t want your own company all the time do you and I like to learn things so get to know different things … (Julie)

Both participants and facilitators commented on how the intervention could provide a feeling of belonging to an inclusive and cohesive group.

I think there’s sort of I dunno whether you call it camaraderie or sort of friendships you could see that sort of developing sort of bonding … (Trevor)

There were several descriptions of people forming friendships and how this brought people together and reduced isolation.

One of them was giving the other one lifts in her car to the group and she said to me, I’ve made a really good new friend there you know, and to think that she only lives round the corner from me and we didn’t even know each other, and now we’ve become friends. (Lynne)

Facilitators commented that friendships were particularly important for participants who had become socially isolated.

They were going to these community things together and they seemed to form quite solid you know foundations for friendships … she was on her own in the flat you know she’d often not see anybody for a long time it’d make her feel down. (Sarah; facilitator)

However, long-term friendships demand a level of compatibility, as observed by this participant who withdrew from the intervention.

I mean when you make friends you usually make friends with somebody who you are compatible with. Don't you? (Liz)

Positive experiences were at variance with other comments about how individual personalities could hinder a group gelling.

We were very very lucky that there were very few personality clashes so, I have made a couple of friends and I know Ann has made several friends from it. (Fred)

Facilitators considered that several individuals needed more time than 16 weeks to get to know others before allowing new people into their space and into their life and some groups took a longer to settle and develop trust between people.

### Sharing Experiences, Expertise, and Solutions

Sharing suggestions on how to overcome barriers to taking part inspired people to adapt their behaviour or situation and “have a go” which could lead to an increased sense of self-worth.

Well it felt nice because, I told them different things … I felt good being able to share my knowledge with other people and it would help them, you know. It’s not, ‘oh that’s nice’, it’s, ‘ooh I’ll try that’, you know … (May)

Both facilitators and participants commented on how sharing stories brought them together.

There was some very good discussions went on in there, elderly discussions which other people wouldn’t have joined in, about our youth and things like that, you know. (Alan)

Participants shared experiences and learning about a variety of subjects. They also talked about sharing personal experiences; eg, of bereavement, mental health, and learning difficulties. However, they also said that they could lose interest if the topic focus was protracted or repeated.

We had quite a lot of sessions reminiscing, about old [the local area]as we remembered it from 50, 60, 70 years ago, which was quite interesting, but you know, once that had been gone through, then the only thing that’s left for you to talk about is your illnesses.(Peter)

Several participants considered that attending the group had given them confidence to share opinions and knowledge on a whole range of issues that were of interest to others.

Yes, I think it was, everybody contributed quite a lot, especially in our group, I cannot obviously comment on other groups, but everyone got involved, everyone was free to, to talk on any subject, free to suggest any activity. I think ours was a brilliant group, we had the right mix and people wanted to be involved and wanted to take part, so yeah that was good …. once we started confiding in each other to a degree, then ideas were passed backward and forwards on how to solve everyday problems in life which was brilliant, yeah. (Fred)

### Practicing Decision Making and Taking the Lead

The facilitators all thought that a unique feature of the intervention was how it fostered independent active choices.

I think with Lifestyle Matters it’s really core to what the programme’s about isn’t it not creating dependency not leading but trying to encourage people to do things for themselves. (Chris; facilitator)

They all commented on how members got involved in running the group in contrast to other psychosocial interventions, which are prescriptive and encourage passivity.

It’s not being done to the group, it’s being done with the group. (Debbie; facilitator)

Nevertheless, participants could be challenged by the notion of taking decisions.

That idea of having choice and kind of power and decision making can take some people a lot longer to kind of get their head around. (Chris; facilitator)

Participants described a democratic approach to decision-making. Facilitator support was important.

Facilitators ran it extremely well and they did open it up very much so that it became the group’s decisions rather than their decisions, which was very good. (Peter)

Constantly handing decision-making over was perceived by facilitators to be a necessary skill for participants and central to their role, but also challenging. They thought that resistance from participants was because it was not what they were used to.

I think traditionally people when they come the groups expect to be in the role of the you know you’re going to tell me … for some people it’s quite a frustrating process.(Sarah; facilitator)

One facilitator said that participants seemed to think that they were incompetent students when they would not make decisions for the group. Another facilitator said it was important that participants did not view them as being health professionals who traditionally make decisions. They identified the importance of being clear about their role and not being risk adverse and over-protective.

### Individual Decision-Making About Lifestyle Choices

Several participants described being able to re-evaluate their lifestyles and make decisions about what and how to change, requiring significant attitudinal change.

What I have realised through that is that its attitude that counts, not age. It’s made me have a real look, sometimes I think I won’t try that because I’m nearly 73 and old people don’t do that, and that’s helped push that idea to the back of my mind again, it’s sort of revitalised me and I’m thinking, ‘well, there are people there 10 years older than me doing the same activities so perhaps I should open my mind and, and increase my boundaries sort of thing, you know. Look to the horizon a little bit more and try more things because you do, as you get older, age becomes a barrier, but you’ve put the barrier in place. It’s not, it’s not what you can’t do it’s what you want to do and that’s how it should be. (Fred)

### Choosing to Make Changes and Being Assertive

Helping participants to gain the necessary confidence to make changes was a theme that ran through all the facilitator interviews; eg, for someone recently bereaved.

They were needing to find things to do by themselves or finding new groups and things to do and they might perhaps have been feeling a little bit down or lacking in confidence to go and do that by themselves before and this has helped with their confidence and things and given them extra connections I suppose which has helped with their well-being, their mental health. (Debbie; facilitator)

Another example was someone developing confidence in computing during individual sessions.

One of the group members he’d never done anything computer before and you know just kind of moving the mouse or just doing very basic things. (Chris; facilitator)

### Sustaining Lifestyle Changes

Nearly all the participants talked about opportunities for groups to continue and action had already been taken to make this happen, eg, swapping telephone numbers, arranging a date to meet or contacting each other with invitations. Many talked about carrying on with activities and exploring new activities. Facilitators said that many but not all of the groups made plans to carry on meeting up as a whole or part of the group.

Group 1 swapped numbers and I think there were pockets of people there that might stay in touch … Group 2 again there were a band that were going to the cinema but it was very different personalities … The group did swap numbers and say they would stay in touch … Group 3 arranged to meet at somebody’s house and I know that happened!

Group 4 had decided to meet in a month’s time at a local pub and maybe do it monthly and see how that went … Group 5 had a meeting arranged to meet for a kind of coffee morning and see how that went and see how many people … group 6 I think again some people will stay in touch but not necessarily as a group … but they have made friendships. (Chris; facilitator)

Missing the intervention was a key driver for continuing to meet. Barriers to continuing included travel problems in rural areas, difficulties identifying when individuals could meet regularly due to other commitments, and a lack of leadership or volunteer organiser. Uncertainty and ambivalence were both expressed.

You know I’m hoping there, very often these things start off enthusiastically and they might sort of taper off a bit. I’m interested to see how long it goes on and hopefully it will go on you know. (John)

Well we’ve all got the phone numbers, but I haven’t heard anything, so I don’t know whether that’s going to carry on, you know, but I would meet up and have coffee if, you know, I’m sure it will do, but this time of year you’ve either got your grandchildren or people’s on holiday and things like that, you know, so I’m sure somewhere along the line we will, you know, sort something out. (Susan)

One group reportedly discussed widening their membership to invite friends who had not attended their group.

One of the facilitators raised ideas for a more gradual process of ending which they thought might be more productive.

We were wondering would it be better if you handed a couple of sessions over where you weren’t there and then went back and just were that bit of support for like, well alright you know, perhaps at week 20, you know, did you meet up and did it work? (Sarah; facilitator)

### Continuing New Friendships

Friendships appeared to motivate people to continue meeting. Certain activities require a companion or are more enjoyable if undertaken with others.

Well I would like to go walking. Yes, I think that would be nice, or perhaps meet and go out for a meal sometimes, you know, maybe things that you’d, you can’t, can’t do on your own or you wouldn’t do on your own. But if you’ve got company you would, you would go. (Betty)

Several participants described striking up and continuing close friendships with one or two individuals with whom they shared common interests.

“I mean I still go to Keep Fit with one of the ladies that was there … so we keep in touch that way … ” (Denise) quote not formatted like the others

Another recounted.

I’ve seen one of the ladies who went to the group, she’s eighty-three and she’s terrific, and she said have you ever been to bingo? So I said oh yes, I says I’m a bit partial to a game of bingo so she said, would you go with me? So I said sure … And we went last Saturday … I didn’t win anything but it were good fun and we nattered and I go and pick her up she likes that. Yes she’s a delightful lady and I’m grateful for the Lifestyle [Matters] just to meet. (May)

Participants invited other group members to go to their favoured activities outside the group; eg, one person invited others to go on a coach trip with her and someone else took a member to their bridge club.

However, not everyone wanted to stay in touch. Group dynamics had a significant effect on the potential for continuation for some, eg, poor group bonding due to lack of common ground.

There was nobody in the group that I, if we’d met outside the group, that I would have said oh … we’ll be friends for life. (Peter)

A second reason was the gender imbalance with some groups having more female members. The intervention failed to bridge this gap with some male participants expressing that they had less opportunity to bond with similar individuals and therefore had not built up friendships to take beyond the 16 weeks.

### Challenges to Participation

How disability can create barriers to involvement in this intervention and in life in general was a universal issue. Nearly all participants described experiencing limitations due to a health condition or age-related disability. However, there was also a strong expressed desire to remain independent.

Well, I declined because I would’ve needed to use my wheelchair (during the out of venue activity) and my wheelchair has to be pushed, you know, it’s not self-motivated. So [facilitator] said, ‘no problem, one of … ’, you know, ‘one of us can help’, and I went, ‘that is the point, I don’t want to go there and spoil somebody else’s day, them pushing me round’, you know. So I said, ‘no I won’t if you don’t mind’. That’s my independence. (May)

All the facilitators observed how graded engagement in activities led to changes in participants’ attitudes towards their ability to be able to participate. An example was provided by a participant who had recently had a double-hip replacement.

She absolutely turned out to be the person who was most gung-ho about trying everything you know from even if she was just sitting down to do things … she’d actually started going to Tai Chi outside of the group and she’d been getting two buses to get there. (Chris; facilitator)

Participants raised two very different attitudes towards age and ageing. Firstly that age in itself is not a barrier to change. Alternatively deteriorating health, disability and/or tiredness meant some participants felt unable to carry out activities they used to do particularly if previous standards had to be compromised as described by this participant who did not continue with the intervention.

… and knitting, sewing, embroidery, you usually dabbled and done things then and usually by the time you’re older oh my eye sights not as good as it was and my concentration isn’t as good as it was and my bodily function you know I just can’t do the things I used to do. (Liz)

Dislike was expressed of discussions that became dominated by ill health, particularly if it was not channelled into potential solutions. Attitude to illness could therefore affect group dynamics.

I suppose it’s inevitable, you are dealing with old people, but in the end it was, who, who was the, who was the more ill … you know like, I have got so and so, “ooh, have you?” “I’ve got that” … oh my god, which was a bit of a downer … It’s, [pauses] there was more I think half full people than half empty. (Peter)

### Hearing Loss

For a small but significant number of participants, hearing loss posed significant problems and the carefully selected group venues could still fail to meet their needs.

I didn’t contribute a lot to it because of my hearing problem and then thinking, no I’ll not saying anything because I can’t, I can’t really hear what other people were saying back to me sort of thing, you know it does hold you back a little, a little bit. (Betty)

The same individual described difficulties following conversations and how this led to frustration and thoughts of withdrawing.

Due to hearing problems some participants were limited to getting to know only the people they sat next to regularly.

So you bond obviously with the person next to you and sort of because I’m deaf … I do have a hearing problem and I must be honest a lot of the things that the group said I didn’t catch particularly those sitting on the other side of the room. (Julie)

If there’s more than one sound everything gets jumbled up. (Susan)

One participant suggested the use of an induction loop. Another wanted a quiet space with no noise from adjacent rooms. Another suggested more controlled chairing of discussions to avoid multiple voices at the same time.

… so somebody’s talking here, somebody’s there, and you need a chairman with a gavel just one at a time please. (John)

Facilitators learnt from the experiences of deaf participants.

We had one lady who’s got a hearing aid in each ear, both ears, she goes to lip-reading classes and she educated us all about these hearing loop systems which I had knew nothing about … we all learnt a lot that day from that lady. (Lynne; facilitator)

Learning about an individual’s hearing condition meant that the rest of the group could respond to this and be more inclusive.

And there was a lady who’d become acutely deaf and she’d been to, on her own back, lip reading lessons, and it, when we realised if you’d gone up to talk, we had to face her because she’d pick up what you were saying … (Alan)

### Finance

The majority of participants considered themselves to be financially comfortable and able to pay for travel and group outings.

There was no one who couldn’t have afforded to throw a fiver up every now and again or something … (Alan)

Nevertheless, the costs associated with improving lifestyle, increasing activity levels, and engaging in the local community were acknowledged. One person raised the potential for embarrassment for those living on a small pension.

When they were saying about going to the pictures and going for a meal yeah that’s fine, nice but what you have got to remember is there’s some people who live just off their pensions and that could’ve been embarrassing because if you go for a meal well it’s gonna, how much is it gonna be? At least ten pound … and the transport, pay to go to the pictures, alright you go certain times you get it cheaper but it’s an added expense … (May)

The facilitators had a different perception in that they perceived Lifestyle Matters to be a low-cost intervention and considered that they had suggested activities that all members could afford. Tips on money saving strategies between participants were encouraged, as was the exploration of free community resources.

### Language and Culture

National identity and language were very important to the Welsh participants. At least half the members of each Welsh group were Welsh first language speakers. Group discussions were held in English with the Welsh-speaking facilitator translating, making it possible for everyone to contribute.

Oh yeah it was quite good because [facilitator name] she’s bilingual so I mean one or two of the older ones you know they prefer some of the stuff in Welsh like which is okay cos I understand it anyway … so I mean you had that mix as well like which is quite good. (Trevor)

When we’ve had people that are struggling to think of what they want to say in English, say it in Welsh and then we’ll figure out what it is you’re trying to say. (Debbie; facilitator)

One perceived advantage of the bi-lingual group sessions from the facilitator perspective was that they fostered community relationships.

You get these localities where you get just English groups doing something or just Welsh groups doing something and what was really really nice actually was to see members of the same community coming together with different first languages and actually really getting on. (Lynne; facilitator)

Bi-lingual groups encouraged English speakers to practice speaking Welsh. It also offered the chance for new perspectives as reported about one participant.

She said she felt restricted in her community some of the things were run by chapel that she just felt were very gossipy and she didn’t want to be a part of but coming to this group had been like a breath of fresh air. (Lynne; facilitator)

The opposing argument was based on a facilitator saying that several Welsh-speaking members expressed a strong preference for the intervention being in Welsh. The importance of wider culture and not just language was emphasised by one of the facilitators.

Personally, even though people did generally get on together very well, I think it is important in such strong Welsh speaking communities to have groups available that are run solely through the medium of Welsh, in terms of language, culture identity, and community, especially in those areas and they should be identified. (Lynne; facilitator)

### Overall Impact of the Intervention

Visits to local community facilities such as art centres and museums encouraged people to continue these activities after intervention cessation. There were several reports from facilitators of people carrying on new activities, eg, Tai Chi, or computer classes, having tried them out.

We have had people saying well we wouldn’t have done it if we weren’t coming to this group. (Sarah; facilitator)

Plans to continue new activities varied amongst participants with the majority stating they would continue. Some thought they had experienced everything already or had little interest, or as one participant illustrated, had not been able to sustain their initial enjoyment.

Well I did try it and I, I got a little office set up, I got bored. (Denise)

When asked directly, the majority of participants felt attending had not had any significant impact on their daily life, but evidence of the intervention as an instigator of behavioural change could be identified when they talked positively about their lifestyle choices, attitude to life, self-awareness and also confidence to try new things which for some was on-going.

No it hasn’t ended. Yes the meetings might have ended but the programme within ourselves is still going on … (May)

## Discussion

Qualitative studies of lifestyle post-retirement illustrate the complex range and interplay of factors, which can influence the nature and course of later life (eg, 20; 21; 22, 23). The aims of this qualitative study were to understand the acceptability, experiences of and short-term impact of an intervention created to enable individuals to positively reframe lifestyle in later life, particularly by understanding the importance of activity for health and well-being.

In the short term, it appears that participation in Lifestyle Matters could assist participants to moderate and in some instances overcome the consequences of age-related illness, disability, and social isolation and make lifestyle changes.

How the benefits described by participants during these interviews mapped onto intervention theory is illustrated in Table 3.

**Table 3:** Mapping Described Benefits to Intervention Theory

	Bandura Mastery	Bandura: Vicarious Experience	Bandura Verbal Persuasion	Bandura Positive States	Law Person	Law Environment	Law Activities
**Benefits described**							
Involving and using locally based facilities	X	X	X	X	X	X	X
Taking the lead	X	X	X	X	X	X	X
Taking decisions	X	X	X	X			
Trying new activities/new learning	X	X	X	X	X	X	X
Sharing activities	X	X	X	X	X	X	X
Enacting lifestyle changes	X	X	X	X	X	X	X
Use of humour				X			
Challenging routines and stereotypes		X	X	X	X	X	X
Meeting different people		X	X	X			
Attitudinal change		X	X	X			
Sustaining change					X	X	X
New friendships				X			
Keeping in touch				X	X	X	X
Positively managing age related disability	X	X	X	X	X	X	X

This shows that certain aspects of the intervention; namely practising decision-making and taking the lead, sharing and enacting activities, and positively managing age-related illness and disability mapped on to all aspects of intervention theory. Succeeding with undertaking new or neglected activities due to verbal support, vicarious observations of others and behavioural modelling based upon those observations was a theme running throughout, which then resulted in more satisfying lifestyle experiences. This is in accord with the conclusions of Bandura,^10^ which are that improved levels of self-efficacy encourage more individual effort and persistence leading to improvements in quality of life. Benefits relating to meeting new people, creating new friendships and sustaining lifestyle change following intervention cessation were only partially fulfilled and engagement in 1:1 sessions, specifically designed to pursue individual goals, was limited. While the overall intervention promotes the re-designing of lifestyle through the identification and enactment of life goals, neither participants nor facilitators described this explicitly. However, there were many implicit examples goal setting and working on goals within the interview data; eg, enacting and sustaining lifestyle changes. Nevertheless, the full extent of sustained behavioural change that the intervention might encourage was unlikely to be achieved due to incomplete delivery of the overall program. Other difficulties, which reportedly eroded ability to be able to gain verbal support from others and model behaviour included hearing loss and language and cultural requirements. In accord with the findings from the interviews at 24 months post intervention, the small numbers of participants who reported deriving significant benefit were those who also had experienced life-changing events and could as a consequence recognize the need to make changes.^16^ Sustainability also has to be considered; the value of multimodal holistic interventions to promote lifestyle change has been noted in other evaluations, but also the need for relapse prevention.^24^

Despite the Lifestyle Redesign intervention demonstrating efficacy in the US^2^,^3^ the trial of the UK adapted intervention found that the intervention had a neutral impact upon quality of life, although there was significant improvement to perceived loneliness.^15^ One potential reason for neutral trial results may be that, although the intervention did have a positive effect on participants, this effect was not great enough to elicit a significant improvement to the trial primary outcome (SF-36 score). Participants recruited to the Lifestyle Matters trial were not necessarily in contact with health or social care services and had a relatively high SF-36 score at baseline.^15^ In contrast, both US studies were conducted with specific populations; the first with people living in low-cost sheltered housing and the second with those already engaged with different forms of day service and who were therefore highly likely to have identified needs. Additionally, as the results of these interviews demonstrate, participants may have required more assistance than was available to them to benefit fully from the intervention and sustain any gains that they may have obtained.

Another issue, which may have compromised trial results is located in the utility of outcome measures for clinical trials.^25^ For example, it has been proposed that generic health outcome questionnaires as such as the SF-36 are open to interpretation and do not identify some important issues when used with older people.^26^ The Better Ageing Project^27^ assessed the effect of a physical-activity programme upon the mental and physical well-being of older adults. Qualitative findings demonstrated some positive improvements, but this was not mirrored by the quantitative trial results. As with Lifestyle Matters, it was deduced that the quantitative measures may have not been sensitive enough to detect change in participants’ well-being and it was also noted that participants were mentally and physically well compared to the intended trial population. In contrast, delivery of a multi-componentmulti component preventive physical health intervention to frail older people aged 80 and over did report short-term positive outcomes in the shorter term, underscoring the importance of intervention targeting.^28^

The Lifestyle Matters trial^15^ highlighted the difficulties of recruiting those most in need of such an intervention; namely people who had become lonely, isolated, and inactive. The previously undertaken feasibility study of the same intervention^6^ recruited those who met these criteria, demonstrating the limits of using recruitment data from feasibility studies to predict recruitment in larger randomized trials where a rapid recruitment drive is usually required,^29^ and in particular recruitment to trials of interventions with a group element such as Lifestyle Matters.

This form of multicomponent intervention is still recommended for practice in the UK^30^ and work continues across the globe to consider how such programmes, designed to allay the effects of age-related decline can be implemented to best effect.^21^,^31^,^32^ This UK-based study was compromised by the study population; the majority were not experiencing decline. The UK challenge is the timely identification of individuals beginning to experience difficulties. Until this is possible, the true benefits of this intervention will not be realised.^33^

## Conclusions

The qualitative evidence presented here demonstrates the positive effect that this intervention could have upon the lives of older people but only if the entire intervention is delivered as intended and it is targeted towards a population who are on the cusp of age-related decline. It also underscores the challenges associated with undertaking large-scale pragmatic trials of complex behavioural interventions.

## Data Sharing Statement

All relevant data are published within this manuscript.
